# Analysis of Body Mass Index in Early and Middle Adulthood and Estimated Risk of Gastrointestinal Cancer

**DOI:** 10.1001/jamanetworkopen.2023.10002

**Published:** 2023-05-10

**Authors:** Holli A. Loomans-Kropp, Asad Umar

**Affiliations:** 1 Division of Cancer Prevention and Control, Department of Internal Medicine, College of Medicine, The Ohio State University, Columbus; 2 Comprehensive Cancer Center, The Ohio State University, Columbus; 3 Gastrointestinal and Other Cancers Research Group, Division of Cancer Prevention, National Cancer Institute, Rockville, Maryland

## Abstract

**Importance:**

In a population with significantly increasing rates of individuals with overweight or obesity, understanding the association of obesity with long-term disease risk, such as cancer, is necessary to improve public health.

**Objective:**

To investigate the association between body mass index (BMI) and gastrointestinal (GI) cancer risk (colorectal cancer [CRC] and noncolorectal GI cancer) in the Prostate, Lung, Colorectal, and Ovarian (PLCO) Cancer Screening Trial.

**Design, Setting, and Participants:**

This retrospective cohort study was a secondary analysis of data from the PLCO Cancer Screening Trial. Participants aged 55 to 74 years were enrolled and randomized to the intervention (screening group) or control group at 10 screening centers between November 8, 1993, and July 2, 2001. The initial analysis of PLCO Cancer Screening Trial data occurred after 13 years of follow-up or December 31, 2009, whichever came first. Participants were reconsented in 2011 and either continued follow-up or refused additional follow-up. For those who reconsented, follow-up for incident cancers continued until December 31, 2014, or death, whichever occurred first. Data analysis for this secondary analysis was performed from April 2022 through November 2022.

**Exposures:**

Body mass index and aspirin use, defined as the frequency of use of aspirin or aspirin-containing substances in the last 12 months.

**Main Outcomes and Measures:**

The primary outcomes were the diagnoses of CRC and noncolorectal GI cancer. The association between BMI and cancer (CRC and noncolorectal GI cancer) was assessed using Cox proportional hazards regression modeling. The association between cancer risk and change in BMI was further analyzed at different ages, and an exploratory analysis was performed to evaluate GI cancer risk among aspirin users.

**Results:**

This analysis included 135 161 participants (median [range] age, 62 [55-78] years; 67 643 [50.0%] female). Overweight BMI in early adulthood (hazard ratio [HR], 1.23; 95% CI, 1.10-1.37) and overweight BMI in middle adulthood (HR, 1.23; 95% CI, 1.13-1.34) and later adulthood (HR, 1.21; 95% CI, 1.10-1.32) as well as obese BMI in middle adulthood (HR, 1.55; 95% CI, 1.38-1.75) and later adulthood (HR, 1.39; 95% CI, 1.25-1.54) were associated with increased risk of CRC. Similar results were observed for the association with overall GI and non-CRC GI risk and BMI in middle and later adulthood. Maintaining overweight or obese BMI or increasing BMI to overweight or obese in later adulthood was also associated with increased CRC risk. Aspirin use 3 or more times per week did not significantly modify this association.

**Conclusions and Relevance:**

In this secondary analysis of the PLCO Cancer Screening Trial, overweight and obese BMI in early and middle adulthood was associated with an elevated risk of CRC and noncolorectal GI cancers. The results of the current study prompt further exploration into the mechanistic role of obese BMI in carcinogenesis.

## Key Points

**Question:**

Is body mass index (BMI) or changing BMI associated with risk of gastrointestinal cancer?

**Findings:**

This cohort study, which used data from the Prostate, Lung, Colorectal, and Ovarian Cancer Screening Trial, found that overweight and obese BMI in early and middle adulthood was associated with an increased risk of gastrointestinal cancer. Maintaining or increasing overweight or obese BMI over time was also associated with an increased risk of gastrointestinal cancer.

**Meaning:**

These findings suggest that overweight and obese BMI over time may increase one’s risk of gastrointestinal cancer.

## Teaser

This cohort study of Prostate, Lung, Colorectal, and Ovarian Cancer Screening Trial data examined the association between gastrointestinal cancer and body mass index in adulthood and the association between changing body mass index and cancer risk.

## Introduction

Colorectal cancer (CRC) is the third most incident cancer among men and women in the US.^1^ Although improvements in CRC detection and screening have shifted CRC diagnosis to more localized and regional disease, a steadily decreasing but still staggering number of incident CRC cases are diagnosed annually.^1^ This may be due to a concurrent increase in risk factors for gastrointestinal (GI) cancer development. Of particular interest, obesity rates are increasing globally.^2^ Obesity is associated with numerous negative outcomes, including the development of type 2 diabetes and other metabolic disorders; cardiovascular diseases, such as hypertension and stroke; and cancer.^3,4,5,6,7^ The World Cancer Research Fund and the International Agency for Cancer Research have estimated that approximately 20% of cancers may be attributed to excess weight gain.^8,9,10^ Gastrointestinal cancers have been strongly associated with obesity, likely because of persistent, chronic inflammation attributable to obesity.^11,12^ Chronic inflammation has been shown to be associated with increased risk of several GI cancers, such as pancreatic (pancreatitis), esophageal (esophagitis and Barrett esophagus), and colorectal (ulcerative colitis and Crohn disease). Epidemiological studies have consistently demonstrated increased GI cancer risk among individuals with overweight and obesity.^13^ Furthermore, an analysis^14^ of the Cancer Prevention Study II found that the risk of GI cancer–specific mortality increased 1.86 to 4.52 among men with obesity and 1.46 to 2.76 times among women with obesity compared with individuals with normal body mass index (BMI [calculated as weight in kilograms divided by height in meters squared]) (18.5-24.9–times increase).

The role of inflammation in cancer dates to observations by Virchow^15^ and was expanded on by Dvorak.^16^ Inflammation can be ascribed to several means, including chronic infection or conditions that result in enhanced proinflammatory signaling, such as obesity. However, many questions remain regarding the impact of heightened baseline inflammation attributed to obesity on cancer risk, such as the effect of obesity or weight gain in early life on later cancer risk or how changing BMI over time alters cancer risk. Recently, the DACHS (Darmkrebs: Chancen der Verhütung durch Screening [Colorectal Cancer: Chances for Prevention Through Screening]) study, a case-control study that evaluated risk factors and screening practices for CRC, found that obesity in early adulthood was associated with increased CRC risk, suggesting that early life events impact later health outcomes.^17^ Previous assessments^18,19^ of BMI in the Prostate, Lung, Colorectal, and Ovarian (PLCO) Cancer Screening Trial found an increased risk of mortality with a 5% or greater increase in BMI, and a separate analysis^20^ found that adenoma and CRC risk was associated with increasing BMI trajectories. A meta-analysis^21^ of prospective studies investigating BMI found a pooled relative risk of 1.33 (95% CI, 1.25-1.42) comparing obese with normal BMI and 1.46 (95% CI, 1.33-1.60) comparing highest with lowest categories of waist circumference. Although the literature has established the precedent that BMI influences CRC risk, much still needs to be explored. Therefore, in the current study, we evaluated the association between GI cancer, CRC, and noncolorectal GI cancer risk and BMI at early, middle, and later adulthood, as well as the association between changing BMI and cancer risk.

## Methods

This cohort study was a secondary analysis of the PLCO Cancer Screening Trial, a large, multicenter randomized clinical trial that evaluated the efficacy of prostate, lung, colorectal, and ovarian cancer screening examinations in reducing mortality. The PLCO Cancer Screening Trial was approved by the institutional review boards of all study sites. Participants provided written informed consent for the original and ancillary studies. Additional approval for the current study was not required because data use in ancillary studies was included in the original consent and all data were deidentified. This study adhered to the Strengthening the Reporting of Observational Studies in Epidemiology (STROBE) reporting guideline.^22^

### Study Design

The PLCO Cancer Screening Trial study design has been described elsewhere.^23,24,25^ Briefly, participants aged 55 to 74 years were enrolled and randomized to the intervention (screening group) or control group at 10 screening centers (University of Alabama at Birmingham, Georgetown University, University of Pittsburgh, Washington University in St Louis, University of Utah, University of Colorado, University of Minnesota, Pacific Health Research and Education Institute [Hawaii], the Henry Ford Health System [Detroit, Michigan], and Marshfield Clinic Research Foundation [Marshfield, Wisconsin]) between November 8, 1993, and July 2, 2001. Exclusion criteria pertinent to the current analysis were age younger than 55 or older than 74 years at the time of randomization; a history of prostate, lung, colorectal, or ovarian cancer; prior surgical removal of the colon; treatment for cancer other than basal or squamous cell carcinoma of the skin; participation in another cancer screening or cancer primary prevention trial; beginning in April 1995, receipt of a colonoscopy, sigmoidoscopy, or barium enema in the 3 years before enrollment; and unwillingness or inability to sign a consent form. Additional PLCO Cancer Screening Trial exclusion criteria can be found elsewhere.^25^ Participants randomized to the intervention group received screening for prostate, lung, colorectal, and ovarian cancers in the designated study years, whereas participants in the control group received standard care.

To complete the current analysis, we implemented additional exclusion criteria. Participants were excluded from the final analysis for the following reasons: (1) no valid baseline questionnaire (BQ), (2) BMI or aspirin use information was incomplete, (3) any personal history of cancer, and (4) discordant responses between the BQ and supplemental questionnaire (SQ), if the SQ was completed and valid and responses were used for analysis (Figure). A BQ was completed on or soon after enrollment. The SQ was distributed to study participants between 2006 and 2008, although completion of the SQ was not required. Both questionnaires are publicly available.^26,27^ Age-specific BMI was calculated using self-reported height and weight at the designated ages from the BQ. In the current analysis, BMI at the age of 20 years is considered early adulthood, BMI at 50 years is considered middle adulthood, and BMI at the time of the study is considered later adulthood, as this refers to individuals 55 years or older. Specifically, the questions used to calculate BMI were as follows: “What is or was your weight at these ages? (Enter the weight in pounds.),” with response categories for weight at 50 years of age, 20 years of age, and current weight (at BQ); and “How tall are you? (Record your height in feet and inches.).” Body mass index was calculated and categorized according to the World Health Organization standard categorization: underweight (BMI <18.5), normal (BMI of 18.5-24.9), overweight (BMI of 25.0-29.9), and obesity (BMI ≥30).^28^ The exploratory analysis used the following questions regarding aspirin use from the BQ and SQ, respectively: “During the last 12 months, have you regularly used aspirin or aspirin-containing products, such as Bayer, Bufferin, or Anacin (Please do not include aspirin-free products such as Tylenol or Panadol)?” and “During the last 12 months, about how often did you usually take aspirin (examples of aspirin include Bayer, Bufferin, Anacin, and baby aspirin)?” Participants who reported aspirin use 3 or more times per week were used for the final analysis, a threshold previously established.^29^

**Figure.**
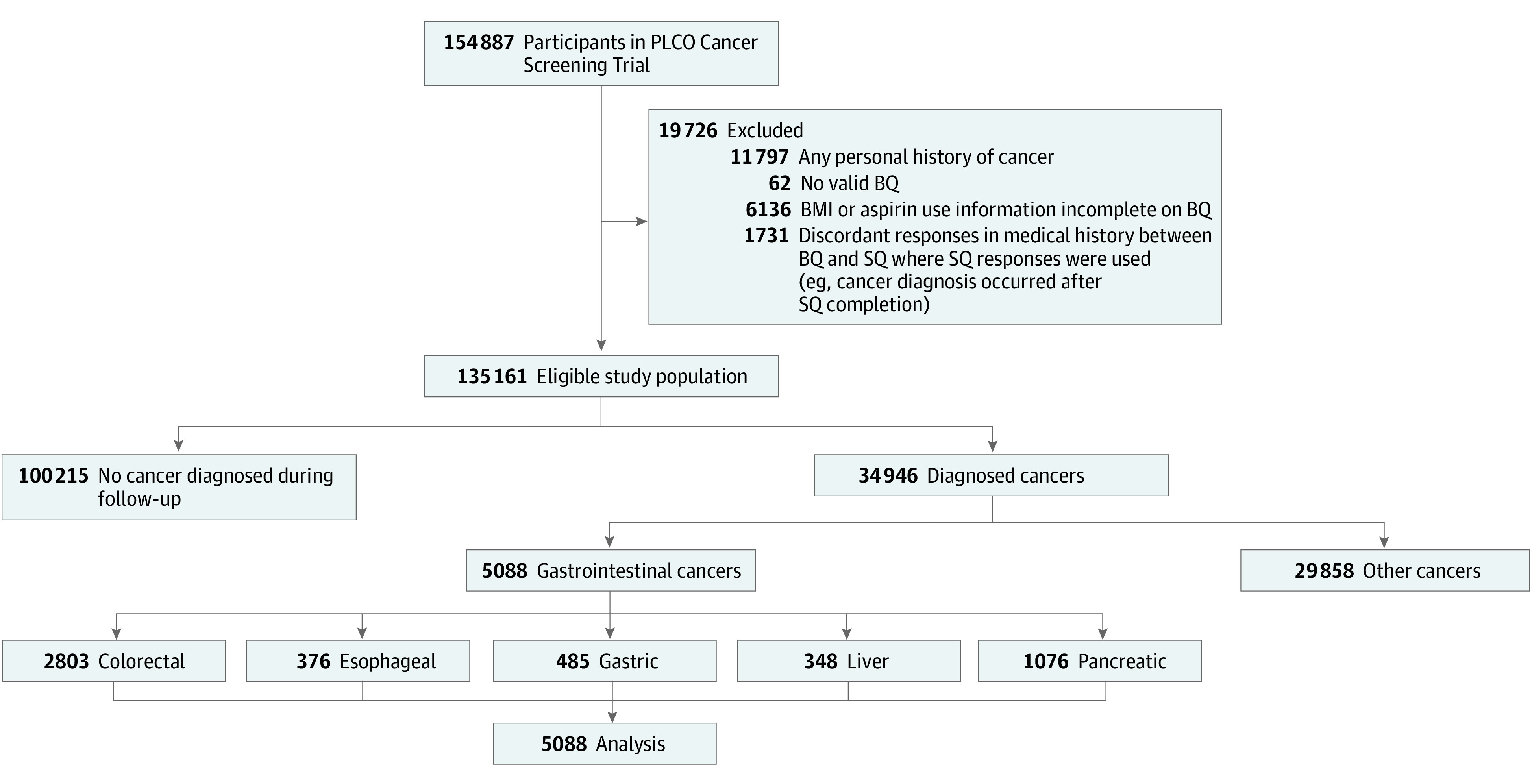
Flowchart of Eligible Prostate, Lung, Colorectal, and Ovarian (PLCO) Cancer Screening Trial Participants for the Study Analysis The final cohort included participants who were older than 55 years with completed information to calculate body mass index (BMI) and aspirin frequency information. BQ indicates baseline questionnaire; SQ, supplemental questionnaire.

The initial analysis of PLCO Cancer Screening Trial data occurred after 13 years of follow-up or December 31, 2009, whichever came first.^23^ Participants reconsented in 2011 and either continued follow-up or refused additional follow-up. For those who reconsented, follow-up for incident cancers continued until December 31, 2014, or death, whichever occurred first. If the incident cancer occurred after reconsent, incident cancers were determined by state cancer registry linkage. The mean (SD) follow-up of the included cohort was 13.9 (6.0) years, and the median (range) follow-up was 14.9 (0-24.2) years. Data analysis for this secondary analysis was performed from April 2022 through November 2022.

### Cancer Incidence

The goal of this analysis was to evaluate the association between BMI and GI cancer risk, separating CRC or noncolorectal GI cancer incidence in the PLCO Cancer Screening Trial. We defined an incident cancer as the first cancer diagnosed during follow-up. Diagnostic information on incident cancers was collected and recorded using an approved medical record abstraction form. *International Classification of Diseases for Oncology, Second Edition* (*ICD-O-2*) codes and diagnosis dates were collected for nonstudy cancer, whereas additional information, such as stage and grade, was collected for study cancers. Incident GI cancers were CRC (codes 153 and 154), esophageal (code 150), gastric (code 151), liver (code 155), and pancreatic (code 157) cancers, identified by *International Classification of Diseases, Ninth Revision* (*ICD-9*) codes (Figure). Follow-up time began at the time of randomization and continued until the date of cancer diagnosis, participant death, or the end of study follow-up. Individuals diagnosed with cancer not included in a given analysis were censored at the time of diagnosis to account for competing risks.

### Statistical Analysis

Time-dependent Cox proportional hazards regression models with competing risks were used to calculate hazard ratios (HRs) and 95% CIs, assessing the associations between BMI and GI cancer (CRC and noncolorectal GI). Univariable regression modeling for risk of CRC and noncolorectal GI cancer was performed to determine variables for inclusion in the multivariable model (eTable 1 in Supplement 1). Variables with a univariable *P* < .05 were included in the final model. Covariates included in the final regression model were age at randomization, study randomization group (intervention or control), study center (University of Colorado, Georgetown University, Pacific Research and Education Institute [Hawaii], Henry Ford Health System, University of Minnesota, Washington University in St Louis, University of Pittsburgh, University of Utah, Marshfield Clinic Research Foundation [Wisconsin], or University of Alabama at Birmingham), sex (male or female), self-reported race and ethnicity (Asian, Hispanic, or Pacific Islander [grouped because the number of participants for each group was small and not appropriately powered to generate an estimate], non-Hispanic Black, and non-Hispanic White), smoking status (never, current, or former), ibuprofen use (<3 or ≥3 times per week), aspirin use (0-<1 time per month, 1-3 times per month, 1-2 times per week, or ≥3 times per week), and history of myocardial infarction, stroke, hypertension, or diabetes. In univariable analyses, race and ethnicity were statistically significant and therefore included in the model. Smoking status, ibuprofen use, aspirin use, and history of myocardial infarction, stroke, hypertension, and diabetes were incorporated into the models as time dependent. The Cochran-Armitage test was used to test for the underlying pattern between the BMI categories. All statistical analyses were performed using SAS software, version 9.4 (SAS Institute Inc). *P* values were 2-tailed, and statistical significance was set at *P* < .05.

## Results

Of 154 887 participants enrolled in the PLCO Cancer Screening Trial, 135 161 (median [range] age, 62 [55-78] years; 8726 [6.5%] Asian, Hispanic, or Pacific Islander; 6920 [5.1%] non-Hispanic Black; 119 453 [88.4%] non-Hispanic White; 62 [0.1%] missing race; 67 643 [50.0%] female and 67 518 [50.0%] male) were included in the analysis. The mean (SD) follow-up time of the eligible cohort was 13.9 (6.0) years, and the median (range) was 14.9 (0-24.2) years. During follow-up, 34 946 (25.9%) were diagnosed with cancer, with 5088 (14.6%) GI cancers. A total of 2803 (55.1%) of the incident GI cancers were CRC, with 376 esophageal cancers (7.4%), 485 gastric cancers (9.5%), 348 liver cancers (6.8%), and 1076 pancreatic cancers (21.1%). The demographic characteristics of the analyzed cohort are included in Table 1, and cancer characteristics are given in eTable 2 in Supplement 1. We modeled BMI at (1) early adulthood (BMI at 20 years of age), (2) middle adulthood (BMI at 50 years of age), and (3) later adulthood (BMI at ≥55 years of age) as both categorical and continuous variables to evaluate the association between BMI and GI cancer risk. Increased risk of overall GI cancer was observed among individuals with overweight (early adulthood: HR, 1.17; 95% CI, 1.08-1.27; middle adulthood: HR, 1.18; 95% CI, 1.11-1.26; later adulthood: HR, 1.17; 95% CI, 1.09-1.25) and obesity (early adulthood: HR, 1.31; 95% CI, 1.08-1.59; middle adulthood: HR, 1.50; 95% CI, 1.37-1.64; later adulthood: HR, 1.38; 95% CI, 1.27-1.49) in early, middle, and later adulthood (Table 2). We observed an increased risk of CRC for individuals with overweight BMI (HR, 1.23; 95% CI, 1.10-1.37) in early adulthood, overweight (HR, 1.23; 95% CI, 1.13-1.34) and obese (HR, 1.55; 95% CI, 1.38-1.75) BMI in middle adulthood, and overweight (HR, 1.21; 95% CI, 1.10-1.32) and obese (HR, 1.39; 95% CI, 1.25-1.54) BMI in later adulthood. Similarly, increased risk of noncolorectal GI cancer was associated with obese BMI (HR, 1.37; 95% CI, 1.04-1.80) in early adulthood, overweight (HR, 1.13; 95% CI, 1.03-1.24) and obese (HR, 1.44; 95% CI, 1.27-1.65) BMI in middle adulthood, and overweight (HR, 1.13; 95% CI, 1.03-1.24) and obese (HR, 1.36; 95% CI, 1.21-1.53) BMI in later adulthood. Individual non-GI cancer risk estimates are included in eTable 3 in Supplement 1. When modeled continuously, we observed 2% to 4% increased risk of both CRC and noncolorectal GI cancer with each 1-unit increase in BMI across all time points (eTable 4 in Supplement 1).

**Table 1.** Demographic Characteristics of the Eligible Prostate, Lung, Colorectal, and Ovarian Cancer Study Trial Population^a^

Characteristic	Total cohort (N = 135 161)	CRC (n = 2803)	Noncolorectal GI cancer (n = 2285)^b^
Age, median (range), y			
Randomization	62 (55-78)	64 (55-74)	64 (55-74)
Diagnosis	NA	71.3 (55.1-95.4)	72.9 (55.1-92.9)
Race and ethnicity			
Hispanic, Asian, or Pacific Islander^c^	8726 (6.5)	185 (6.6)	251 (11.0)
Non-Hispanic Black	6920 (5.1)	149 (5.3)	122 (5.3)
Non-Hispanic White	119 453 (88.4)	2469 (88.1)	1912 (83.7)
Missing	62 (0.1)	0	0
Randomization group			
Intervention	68 452 (50.6)	1282 (45.7)	1147 (50.2)
Control	66 709 (49.4)	1521 (54.3)	1138 (49.8)
Center			
University of Colorado	11 614 (8.6)	229 (8.2)	215 (9.4)
Georgetown University	6214 (4.6)	119 (4.3)	105 (4.6)
Pacific Research and Education Institute (Hawaii)	9261 (6.9)	233 (8.3)	265 (11.6)
Henry Ford Health System	21 726 (16.1)	333 (11.9)	218 (9.5)
University of Minnesota	23 840 (17.6)	581 (20.7)	472 (20.7)
Washington University in St Louis	13 619 (10.1)	316 (11.3)	221 (9.7)
University of Pittsburgh	15 545 (11.5)	372 (13.3)	360 (15.8)
University of Utah	13 619 (9.7)	194 (6.9)	110 (4.8)
Marshfield Clinic Research Foundation	14 650 (10.8)	331 (11.8)	255 (11.2)
University of Alabama at Birmingham	5627 (4.2)	95 (3.4)	64 (2.8)
Sex			
Male	67 518 (50.0)	1614 (57.6)	1558 (68.2)
Female	67 643 (50.0)	1189 (42.4)	727 (31.8)
Smoking status			
Never	62 597 (46.3)	1147 (40.9)	823 (36.0)
Current	14 548 (10.8)	336 (12.0)	365 (16.0)
Former	58 001 (42.9)	1330 (47.4)	1096 (48.0)
Missing	15 (0.0)	0 (0.0)	1 (0.0)
History of myocardial infarction			
No	124 622 (92.2)	2547 (90.9)	2043 (89.4)
Yes	9828 (7.1)	244 (8.7)	235 (10.3)
Missing	711 (0.8)	12 (0.4)	8 (0.3)
History of stroke			
No	131 583 (97.3)	2733 (97.5)	2224 (97.3)
Yes	2918 (2.2)	59 (2.2)	53 (2.3)
Missing	660 (0.5)	11 (0.3)	8 (0.4)
History of hypertension			
No	90 496 (67.0)	1870 (66.7)	1398 (61.2)
Yes	44 039 (32.6)	922 (32.9)	883 (38.6)
Missing	626 (0.4)	11 (0.4)	4 (0.2)
History of diabetes			
No	124 514 (92.1)	2531 (90.3)	2003 (87.7)
Yes	9980 (7.4)	259 (9.2)	277 (12.1)
Missing	667 (0.5)	13 (0.5)	5 (0.3)
BMI category			
Underweight (BMI <18.5)	1016 (0.8)	18 (0.6)	16 (0.7)
Normal (BMI of 18.5-24.9)	45 213 (33.5)	847 (30.2)	667 (29.2)
Overweight (BMI of 25.0-29.9)	57 256 (42.4)	1257 (44.8)	1023 (44.8)
Obesity (BMI ≥30.0)	31 676 (23.4)	681 (24.3)	579 (25.3)
Aspirin use frequency			
None/<1 time/mo	70 012 (51.8)	1510 (53.9)	1136 (49.7)
1-2 times/mo	13 469 (10.0)	292 (10.4)	213 (9.3)
1-2 times/wk	6172 (4.6)	116 (4.1)	95 (4.2)
≥3 times/wk	45 508 (33.7)	885 (31.6)	841 (36.8)
Ibuprofen use			
No	97 006 (71.8)	2087 (74.8)	1720 (75.3)
Yes	37 635 (27.8)	708 (25.2)	556 (24.3)
Missing	520 (0.4)	8 (0.4)	9 (0.4)

Abbreviations: BMI, body mass index (calculated as weight in kilograms divided by height in meters squared); CRC, colorectal cancer; GI, gastrointestinal; NA, not applicable.

^a^ Data are presented as number (percentage) of patients unless otherwise indicated.

^b^ Noncolorectal GI cancer includes esophageal, gastric, liver, and pancreatic cancers.

^c^ Hispanic, Asian, and Pacific Islander participants were reported in aggregate because of small group numbers.

**Table 2.** Multivariable Analysis of Cancer Risk by Categorical BMI in Early, Middle, and Later Adulthood^a^

BMI	HR (95% CI)
**Gastrointestinal cancer incidence**	
Early adulthood	
<18.5	0.97 (0.87-1.08)^b^
18.5-24.9	1 [Reference]
25.0-29.9	1.17 (1.08-1.27)
≥30.0	1.31 (1.08-1.59)
Middle adulthood	
<18.5	1.08 (0.79-1.49)^b^
18.5-24.9	1 [Reference]
25.0-29.9	1.18 (1.11-1.26)
≥30.0	1.50 (1.37-1.64)
Later adulthood	
<18.5	1.05 (0.75-1.48)^b^
18.5-24.9	1 [Reference]
25.0-29.9	1.17 (1.09-1.25)
≥30.0	1.38 (1.27-1.49)
**Colorectal cancer incidence**	
Early adulthood	
<18.5	1.00 (0.87-1.15)^b^
18.5-24.9	1 [Reference]
25.0-29.9	1.23 (1.10-1.37)
≥30.0	1.25 (0.95-1.68)
Middle adulthood	
<18.5	1.10 (0.73-1.66)^b^
18.5-24.9	1 [Reference]
25.0-29.9	1.23 (1.13-1.34)
≥30.0	1.55 (1.38-1.75)
Later adulthood	
<18.5	0.97 (0.61-1.55)^b^
18.5-24.9	1 [Reference]
25.0-29.9	1.21 (1.10-1.32)
≥30.0	1.39 (1.25-1.54)
**Noncolorectal gastrointestinal cancer incidence**	
Early adulthood	
<18.5	0.93 (0.78-1.10)^c^
18.5-24.9	1 [Reference]
25.0-29.9	1.11 (0.99-1.25)
≥30.0	1.37 (1.04-1.80)
Middle adulthood	
<18.5	1.07 (0.65-1.75)^b^
18.5-24.9	1 [Reference]
25.0-29.9	1.13 (1.03-1.24)
≥30.0	1.44 (1.27-1.65)
Later adulthood	
<18.5	1.17 (0.71-1.93)^b^
18.5-24.9	1 [Reference]
25.0-29.9	1.13 (1.02-1.24)
≥30.0	1.36 (1.21-1.53)

Abbreviations: BMI, body mass index (calculated as weight in kilograms divided by height in meters squared); HR, hazard ratio.

^a^ Body mass index at the age of 20 years is considered early adulthood, BMI at the age of 50 years is considered middle adulthood, and BMI at the time of the study is considered later adulthood, as refers to individuals 55 years or older. The model was adjusted for age at randomization, randomization arm, center, sex, race, smoking status, aspirin use, ibuprofen use, and history of myocardial infarction, stroke, hypertension, or diabetes.

^b^ *P* < .001 for trend.

^c^ *P* < .01 for trend.

We next wanted to investigate whether changing BMI over time differentially influenced CRC and noncolorectal GI cancer risk, particularly if the participants changed BMI categories, for example, moving from normal BMI in early adulthood to overweight in later adulthood (Table 3). We found that individuals who had overweight or obese BMI in early and later adulthood (HR, 1.45; 95% CI, 1.28-1.64; *P* < .001) and those who moved from underweight or normal BMI in early adulthood to overweight or obese BMI in later adulthood (HR, 1.23; 95% CI, 1.13-1.34; *P* < .001) had increased risk of CRC. A similar pattern was observed for noncolorectal GI cancer risk (no change in overweight or obese BMI: HR, 1.29; 95% CI, 1.13-1.47; *P* < .001; underweight or normal to overweight or obese BMI: HR, 1.17; 95% CI, 1.06-1.29; *P* = .002). When we modeled change in BMI from middle to later adulthood, we found that consistent overweight or obese BMI (HR, 1.37; 95% CI, 1.25-1.51; *P* < .001), changing from overweight or obese to underweight or normal BMI (HR, 1.47; 95% CI, 1.21-1.78; *P* < .001), and changing from underweight or normal to overweight or obese BMI (HR, 1.20; 95% CI, 1.06-1.34; *P* = .003) were associated with increased risk of CRC. Statistical significance was only observed for static overweight or obesity between middle and later adulthood and noncolorectal GI cancer risk (HR, 1.25; 95% CI, 1.12-1.38; *P* < .001). These data suggest that alterations in BMI over time may influence one’s risk of GI cancer.

**Table 3.** Multivariable Analysis of Cancer Risk by Change in BMI

BMI change	Total No. of cases	CRC			Noncolorectal GI cancer		
		No. of cases	HR (95% CI)^a^	*P* value	No. of cases	GI HR (95% CI)^a^	*P* value
**Change in BMI from early to later adulthood**							
No change in underweight or normal	44 133	817	1 [Reference]	NA	642	1 [Reference]	NA
No change in overweight or obesity	18 581	456	1.45 (1.28-1.64)	<.001	391	1.29 (1.13-1.47)	<.001
Overweight or obesity to underweight or normal	1673	38	1.13 (0.81-1.57)	.47	32	1.10 (0.77-1.57)	.60
Underweight or normal to overweight or obesity	69 749	1470	1.23 (1.13-1.34)	<.001	1197	1.17 (1.06-1.29)	.002
**Change in BMI from middle to later adulthood**							
No change in underweight or normal	41 588	744	1 [Reference]	NA	590	1 [Reference]	NA
No change in overweight or obesity	66 099	1459	1.37 (1.25-1.51)	<.001	1240	1.25 (1.12-1.38)	<.001
Overweight or obesity to underweight or normal	4497	120	1.47 (1.21-1.78)	<.001	92	1.22 (0.98-1.52)	.08
Underweight or normal to overweight or obesity	22 458	471	1.20 (1.06-1.34)	.003	354	1.14 (0.99-1.30)	.06

Abbreviations: BMI, body mass index (calculated as weight in kilograms divided by height in meters squared); CRC, colorectal cancer; GI, gastrointestinal; HR, hazard ratio; NA, not applicable.

^a^ The model was adjusted for age at randomization, randomization arm, center, sex, race, smoking status, aspirin use, ibuprofen use, and history of myocardial infarction, stroke, hypertension, or diabetes.

Finally, because of the potential influence of overweight or obese BMI on cancer preventive agent efficacy, we wanted to evaluate the association between BMI and CRC and noncolorectal GI cancer risk among frequent aspirin users. Among frequent aspirin users, overweight or obese BMI in early (HR, 1.44; 95% CI, 1.23-1.68; *P* < .001), middle (HR, 1.45; 95% CI, 1.26-1.66; *P* < .001), and later (HR, 1.43; 95% CI, 1.24-1.65; *P* < .001) adulthood was associated with increased risk of CRC (eTable 5 in Supplement 1). Similar associations were observed with noncolorectal GI cancer risk.

## Discussion

In this cohort study, we found that overweight and obese BMI at different ages and change in BMI over time may be associated with increased risk of GI cancers. We further found that aspirin use 3 or more times per week did not modify this association. Aspirin use for cancer prevention has been well supported by decades of epidemiological evidence. Previous secondary analyses^29,30^ demonstrated the efficacy of aspirin in reducing the risk of CRC and bladder cancer mortality. However, the impact of BMI on this association has not been adequately delineated. Furthermore, the updated US Preventive Services Task Force recommendations for aspirin use to prevent cardiovascular disease discusses the withdrawal of aspirin use for CRC prevention, of which it had previously been given a B rating for individuals aged 50 to 69 years with a 10% or greater risk of cardiovascular disease, citing insufficient or conflicting evidence.^31,32,33^

Obesity results from the buildup and storage of white adipose tissue, or fat. Adipose cells can induce the inflammatory response and promote immune cell dysfunction through the secretion of adipokines and proinflammatory cytokines, leading to further downstream mechanistic dyregulation.^34^ Individuals with obesity are at higher risk of several conditions, including cancer. Interestingly, not all cancers are significantly associated with obesity; rather, it is more limited to those where cancer cells grow near adipose cells, potentially due to the impact of adipose cells on tumorigenesis.^35^ Research has indicated significant crosstalk between cancer cells and adipocytes. For example, in vitro CRC cell line coculture with adipocytes has demonstrated increased cancer cell proliferation, migration, and nutrient transfer (eg, ketones and fatty acids) from adipocytes to the cancer cells.^36^ Transcriptomic analysis of the ColoCare Study, a prospective cohort of newly diagnosed CRCs, found enrichment of pathways, such as fibrosis and glycolytic metabolism, associated with adipose–tumor tissue crosstalk.^37^ Similar findings have been observed for noncolorectal GI cancers.^38,39,40^ Although likely not the initiator, excess adipocytes promote tumorigenesis through supplying cancer cells with much-needed nutrients and stimulation of oncogenic pathways. Therefore, cancer prevention mechanisms that target the harmful physiologic effects of obesity may work to counteract tumorigenesis.

As found in the current study, obesity may alter the cancer preventive effect of aspirin. Our results indicate that individuals with overweight and obese BMIs had an increased risk of CRC and noncolorectal GI cancer with aspirin use 3 or more times per week, suggesting that aspirin may not be efficacious for prevention in overweight or obese states. The ability of aspirin to protect against GI cancers may be blunted in people with obesity because of inadequate dosing.^41,42^ A suggestion may be that individuals with obesity need to increase aspirin frequency or dosage; however, increased aspirin use comes with its own risks, such as gastrointestinal bleeding.^43^ In our analysis, we did not account for participant dosing, a noted limitation to the study. Additional studies evaluating the impact of aspirin dose on cancer prevention, accounting for participant BMI or weight gain, are needed to better delineate aspirin’s role. The Cancer Prevention Project 3 (CaPP3) is currently under way to discern the effect of differential aspirin dosing (100, 300, or 600 mg) in a cohort of individuals with Lynch syndrome. The CaPP3 study is ongoing; however, the eventual results of this study may be translatable to the general, average-risk population.

### Limitations

Although the findings from the current study are significant, important limitations should be noted. Despite the strengths of baseline and supplemental information, including detailed BMI information at different ages and extended follow-up with linked participant outcomes, the current study is a secondary analysis of a completed cancer screening trial; therefore, the collection of exposure and outcome information was not included as part of the original study. Additionally, all BQ and SQ information was collected by self-report, which includes height and weight data used to calculate BMI. Therefore, aspirin dosing information was not collected as part of the BQ and was not accounted for in this analysis. In addition, we were unable to correlate changes in BMI with aspirin use. Aspirin use, as stated in the BQ and SQ, was reported during the last year, whereas BMI could have changed at any point before the questionnaires. However, in the current analysis, we were only evaluating the association between these 2 factors, not causation. Finally, it is possible that not all confounders were accounted for in our multivariable logistic regression models.

## Conclusions

This cohort study found increased GI cancer risk among individuals with overweight and obese BMI reported at early, middle, and later adulthood. We also found that increasing BMI over time was associated with increased risk of CRC and noncolorectal GI cancers. This association was not modified by aspirin use 3 or more times per week. The results of the current study prompt further exploration into the mechanistic role of obese BMI in carcinogenesis. Finally, future research must focus on identifying cancer prevention mechanisms for this high-risk group.

## Notes

Supplement 1. **eTable 1.** Univariable Proportional Hazards Models of Colorectal Cancer (CRC) and Non-CRC Gastrointestinal Cancer (GI) Incidence **eTable 2.** Cancer Characteristics by Body Mass Index at Randomization **eTable 3.** Multivariable Analysis of Non-CRC GI Cancer Risk (Liver, Pancreatic, Esophageal, Gastric) by Categorical Body Mass Index (BMI) at Early, Mid- and Later Adulthood **eTable 4.** Colorectal and Non-Colorectal Gastrointestinal Cancer Risk in Early, Mid- and Later Adulthood, Using Body Mass Index (BMI) as a Continuous Variable **eTable 5.** Multivariable Analysis of CRC and Non-CRC GI Cancer Risk by Early, Mid- and Later Adulthood Body Mass Index (BMI) Among Frequent Aspirin Users

Supplement 2.
Data Sharing Statement
