# Supplementary material for: Analysis of Body Mass Index in Early and Middle Adulthood and Estimated Risk of Gastrointestinal Cancer
Source: JAMA Netw Open. 2023 May 10;6(5):e2310002. doi: 10.1001/jamanetworkopen.2023.10002 (PMC10173015; doi:10.1001/jamanetworkopen.2023.10002)
Supplement: Supplement 1. — eTable 1. Univariable Proportional Hazards Models of Colorectal Cancer (CRC) and Non-CRC Gastrointestinal Cancer (GI) Incidence eTable 2. Cancer Characteristics by Body Mass Index at Randomization eTable 3. Multivariable Analysis of Non-CRC GI Cancer Risk (Liver, Pancreatic, Esophageal, Gastric) by Categorical Body Mass Index (BMI) at Early, Mid- and Later Adulthood eTable 4. Colorectal and Non-Colorectal Gastrointestinal Cancer Risk in Early, Mid- and Later Adulthood, Using Body Mass Index (BMI) as a Continuous Variable eTable 5. Multivariable Analysis of CRC and Non-CRC GI Cancer Risk by Early, Mid- and Later Adulthood Body Mass Index (BMI) Among Frequent Aspirin Users [file jamanetwopen-e2310002-s001.pdf]

Supplementary Online Content

Loomans-Kropp HA, Umar A. Analysis of body mass index in early and middle adulthood and estimated risk of gastrointestinal cancer. *JAMA Netw Open*. 2023;6(5):e2310002. doi:10.1001/jamanetworkopen.2023.10002

eTable 1. Univariable Proportional Hazards Models of Colorectal Cancer (CRC) and Non-CRC Gastrointestinal Cancer (GI) Incidence
eTable 2. Cancer Characteristics by Body Mass Index at Randomization
eTable 3. Multivariable Analysis of Non-CRC GI Cancer Risk (Liver, Pancreatic, Esophageal, Gastric) by Categorical Body Mass Index (BMI) at Early, Mid- and Later Adulthood
eTable 4. Colorectal and Non-Colorectal Gastrointestinal Cancer Risk in Early, Mid- and Later Adulthood, Using Body Mass Index (BMI) as a Continuous Variable
eTable 5. Multivariable Analysis of CRC and Non-CRC GI Cancer Risk by Early, Mid- and Later Adulthood Body Mass Index (BMI) Among Frequent Aspirin Users

This supplementary material has been provided by the authors to give readers additional information about their work.

© 2023 Loomans-Kropp HA et al. *JAMA Network Open.*

eTable 1. Univariable Proportional Hazards Models of Colorectal Cancer (CRC) and Non-CRC Gastrointestinal Cancer (GI) Incidence

Time-Varying Covariates Were Used Where Available.

Variable	CRC (N=2803) HR (95%CI)[a,b]	*p*-value	non-CRC GI (N=2285) HR (95%CI)[a,b]	*p*-value
Age at randomization	1.07 (1.06, 1.07)	<0.001	1.06 (1.05, 1.07)	<0.001
Self-reported race				
Hispanic/Asian/PI	0.94 (0.82, 1.10)	0.49	1.61 (1.41, 1.84)	<0.001
Non-Hispanic Black	1.20 (1.01, 1.41)	0.03	1.33 (1.11, 1.60)	0.002
Non-Hispanic White	1.0 (ref)		1.0 (ref)	
Randomization arm				
Intervention	1.0 (ref)		1.0 (ref)	
Control	1.22 (1.13, 1.31)	<0.001	1.03 (0.95, 1.11)	0.54
Center				
University of Colorado	1.0 (ref)		1.0 (ref)	
Georgetown University	1.01 (0.81, 1.26)	0.94	0.95 (0.76, 1.20)	0.69
Pacific Research and Education Institute (Honolulu)	1.17 (0.97, 1.40)	0.10	1.37 (1.14, 1.64)	<0.001
Henry Ford Health System	0.90 (0.77, 1.07)	0.21	0.66 (0.55, 0.80)	<0.001
University of Minnesota	1.19 (1.02, 1.39)	0.02	1.02 (0.87, 1.20)	0.81
Washington University in St. Louis	1.18 (1.00, 1.40)	0.05	0.88 (0.73, 1.07)	0.20
University of Pittsburgh	1.18 (0.99, 1.39)	0.06	1.19 (1.01, 1.41)	0.03
University of Utah	0.85 (0.70, 1.03)	0.09	0.54 (0.43, 0.69)	<0.001
Marshfield Clinic Research Foundation	1.16 (0.98, 1.37)	0.09	0.95 (0.80, 1.14)	0.61
University of Alabama Birmingham	1.02 (0.80, 1.29)	0.90	0.80 (0.60, 1.06)	0.11
Sex				
Male	1.0 (ref)		1.0 (ref)	
Female	0.69 (0.64, 0.74)	<0.001	0.43 (0.40, 0.47)	<0.001
Smoking status				
Never	1.0 (ref)		1.0 (ref)	
Current	1.61 (1.42, 1.82)	<0.001	2.55 (2.24, 2.89)	<0.001
Former	1.24 (1.14, 1.34)	<0.001	1.45 (1.32, 1.59)	<0.001
History of heart attack				
No	1.0 (ref)		1.0 (ref)	
Yes	1.14 (1.01, 1.28)	0.04	1.44 (1.27, 1.63)	<0.001
History of stroke				
No	1.0 (ref)		1.0 (ref)	
Yes	0.68 (0.55, 0.85)	<0.001	0.87 (0.70, 1.08)	0.20

History of hypertension				
No	1.0 (ref)		1.0 (ref)	
Yes	0.76 (0.71, 0.82)	<0.001	1.00 (0.92, 1.08)	0.91
History of diabetes				
No	1.0 (ref)		1.0 (ref)	
Yes	1.01 (0.90, 1.13)	0.93	1.53 (1.37, 1.71)	<0.001
Body mass index category				
<18.5	0.89 (0.69, 1.14)	0.34	0.99 (0.75, 1.28)	0.87
18.5-24.9	1.0 (ref)		1.0 (ref)	
25.0-29.9	1.18 (1.08, 1.29)	<0.001	1.18 (1.07, 1.31)	0.001
≥30.0	1.14 (1.03, 1.26)	0.01	1.21 (1.08, 1.36)	0.001
Aspirin use frequency				
None/<1 time/month	1.0 (ref)		1.0 (ref)	
1-3 times/month	0.84 (0.73, 0.96)	0.01	0.78 (0.66, 0.92)	0.003
1-2 times/week	0.73 (0.60, 0.89)	0.001	0.85 (0.68, 1.05)	0.13
3+ times/week	0.64 (0.59, 0.70)	<0.001	0.80 (0.74, 0.88)	<0.001
Ibuprofen use 3+/week				
No	1.0 (ref)		1.0 (ref)	
Yes	1.29 (1.14, 1.45)	<0.001	1.22 (1.06, 1.41)	0.005

[a]Model adjusted for age at randomization, randomization arm, center, sex, race, smoking status, aspirin use, ibuprofen use, and history of heart attack, stroke, hypertension, or diabetes.

[b]Time-varying variables: smoking status, history of heart attack, history of stroke, history of hypertension, history of diabetes, BMI, aspirin use frequency, ibuprofen use, non-time varying: age at randomization, self-reported race/ethnicity, center, sex

Abbreviations: 95%CI, 95% confidence interval; CRC, colorectal cancer; ref, reference; GI, gastrointestinal cancer; HR, hazard ratio; N, number

© 2023 Loomans-Kropp HA et al. *JAMA Network Open.*

eTable 2. Cancer Characteristics by Body Mass Index at Randomization

	Body mass index	
	**<25.0**	**≥25.0**
**Colorectal cancer**		
Location[a]		
Rectosigmoid	350	769
Splenic flexure/descending	53	128
Proximal	451	1014
Unclear	11	27
Grade[a]		
Well differentiated	69	198
Moderately differentiated	525	1159
Poorly differentiated	143	277
Undetermined	11	28
Unknown	117	276
Clinical stage[b]		
I	249	615
II	225	451
III	205	467
IV	124	267
Cannot be assessed	25	41
Summary stage[a]		
In situ	3	3
Localized	345	794
Regional	271	629
Distant	127	270
Undetermined	119	242
**Non-CRC GI cancer**		
Cancer type[c]		
Liver	84	264
Pancreas	357	719
Esophageal	102	274
Gastric	140	345
Grade[a]		
Well differentiated	33	98
Moderately differentiated	147	313
Poorly differentiated	162	423
Undetermined	8	20
Unknown	333	748

[a]Missing N=53
[b]Missing N=187
[c]Missing N=33

© 2023 Loomans-Kropp HA et al. *JAMA Network Open.*

eTable 3. Multivariable Analysis of Non-CRC GI Cancer Risk (Liver, Pancreatic, Esophageal, Gastric) by Categorical Body Mass Index (BMI) at Early, Mid- and Later Adulthood

Early Adulthood Is BMI At Age 20, Mid-Adulthood Is BMI At Age 50, And Later Adulthood Is BMI At The Age At Study Randomization (≥55 Years).

Liver cancer incidence		
**BMI**	**HR (95% CI)[a]**	***p*-for-trend**
**Early adulthood**		
<18.5	1.06 (0.69, 1.63)	*
18.5-24.9	1.0 (ref.)	
25.0-29.9	1.29 (0.98, 1.69)	
>=30.0	**1.83 (1.02, 3.30)**	
**Mid-adulthood**		
<18.5	NC	***
18.5-24.9	1.0 (ref.)	
25.0-29.9	1.25 (0.97, 1.60)	
>=30.0	**1.77 (1.28, 2.44)**	
**Later adulthood**		
<18.5	1.30 (0.32, 5.27)	***
18.5-24.9	1.0 (ref.)	
25.0-29.9	1.20 (0.91, 1.58)	
>=30.0	**1.91 (1.41, 2.59)**	

Pancreatic cancer incidence		
**BMI**	**HR (95% CI)[a]**	***p*-for-trend**
**Early adulthood**		
<18.5	0.93 (0.74, 1.18)	NS
18.5-24.9	1.0 (ref)	
25.0-29.9	0.99 (0.83, 1.19)	
>=30.0	1.12 (0.72, 1.76)	
**Mid-adulthood**		
<18.5	1.29 (0.71, 2.36)	*
18.5-24.9	1.0 (ref.)	
25.0-29.9	1.13 (0.98, 1.30)	
>=30.0	**1.25 (1.03, 1.53)**	

© 2023 Loomans-Kropp HA et al. *JAMA Network Open.*

**Later adulthood**		
<18.5	1.28 (0.68, 2.41)	NS
18.5-24.9	1.0 (ref.)	
25.0-29.9	1.10 (0.96, 1.27)	
>=30.0	1.16 (0.97, 1.38)	

Esophageal cancer incidence

BMI	HR (95% CI)	*p*-for-trend
**Early adulthood**		
<18.5	1.00 (0.64, 1.56)	NS
18.5-24.9	1.0 (ref.)	
25.0-29.9	1.01 (0.77, 1.33)	
>=30.0	**2.20 (1.28, 3.79)**	
**Mid-adulthood**		
<18.5	2.02 (0.64, 6.36)	**
18.5-24.9	1.0 (ref.)	
25.0-29.9	1.23 (0.97, 1.55)	
>=30.0	**1.81 (1.32, 2.48)**	
**Later adulthood**		
<18.5	1.35 (0.33, 5.49)	*
18.5-24.9	1.0 (ref.)	
25.0-29.9	1.08 (0.84, 1.39)	
>=30.0	**1.47 (1.09, 1.97)**	

Gastric cancer incidence

BMI	HR (95% CI)	*p*-for-trend
**Early adulthood**		
<18.5	0.81 (0.55, 1.20)	*
18.5-24.9	1.0 (ref.)	
25.0-29.9	**1.33 (1.05, 1.68)**	
>=30.0	0.88 (0.41, 1.86)	
**Mid-adulthood**		
<18.5	0.62 (0.15, 2.48)	NS
18.5-24.9	1.0 (ref.)	
25.0-29.9	1.00 (0.81, 1.22)	
>=30.0	**1.38 (1.04, 1.83)**	

© 2023 Loomans-Kropp HA et al. *JAMA Network Open.*

**Later adulthood**		
<18.5	0.71 (0.18, 2.87)	*
18.5-24.9	1.0 (ref.)	
25.0-29.9	1.18 (0.95, 1.47)	
>=30.0	**1.40 (1.08, 1.82)**	

[a]Model adjusted for age at randomization, randomization arm, center, sex, race, smoking status, aspirin use, ibuprofen use, and history of heart attack, stroke, hypertension, or diabetes.
*p<0.05, **p<0.01, ***p<0.001

Abbreviations: 95% CI, 95% confidence interval; BMI, body mass index; HR, hazard ratio; NS, not significant

© 2023 Loomans-Kropp HA et al. *JAMA Network Open.*

eTable 4. Colorectal and Non-Colorectal Gastrointestinal Cancer Risk in Early, Mid- and Later Adulthood, Using Body Mass Index (BMI) as a Continuous Variable

Colorectal cancer incidence

Continuous BMI	HR (95%CI)[a]	*p*-value
Early adulthood	1.02 (1.01, 1.04)	<0.001
Mid-adulthood	1.04 (1.03, 1.05)	<0.001
Later adulthood	1.03 (1.02, 1.04)	<0.001

Non-colorectal cancer incidence

Continuous BMI	HR (95%CI)[a]	*p*-value
Early adulthood	1.03 (1.01, 1.04)	<0.001
Mid-adulthood	1.03 (1.02, 1.04)	<0.001
Later adulthood	1.02 (1.01, 1.03)	<0.001

[a]Model adjusted for age at randomization, randomization arm, center, sex, race, smoking status, aspirin use, ibuprofen use, and history of heart attack, stroke, hypertension, or diabetes.

Abbreviations: 95% CI, 95% confidence interval; BMI, body mass index; HR, hazard ratio

© 2023 Loomans-Kropp HA et al. *JAMA Network Open.*

eTable 5. Multivariable Analysis of CRC and Non-CRC GI Cancer Risk by Early, Mid- and Later Adulthood Body Mass Index (BMI) Among Frequent Aspirin Users

Aspirin Use 3+ times/week (N=61110)	BMI	Hazard Ratio (95% CI)[a]	*p-value*
Early adulthood BMI			
**Colorectal cancer risk**			
	Underweight/normal	1.0 (ref.)	
	Overweight/obese	**1.44 (1.23, 1.68)**	<0.001
**Non-colorectal cancer risk**			
	Underweight/normal	1.0 (ref.)	
	Overweight/obese	**1.18 (1.01, 1.39)**	0.04
Mid-adulthood BMI			
**Colorectal cancer risk**			
	Underweight/normal	1.0 (ref.)	
	Overweight/obese	**1.45 (1.26, 1.66)**	<0.001
**Non-colorectal cancer risk**			
	Underweight/normal	1.0 (ref.)	
	Overweight/obese	**1.21 (1.05, 1.39)**	0.009
Later adulthood (≥55 years)			
**Colorectal cancer risk**			
	Underweight/normal	1.0 (ref.)	
	Overweight/obese	**1.43 (1.24, 1.65)**	<0.001
**Non-colorectal cancer risk**			
	Underweight/normal	1.0 (ref.)	
	Overweight/obese	1.16 (1.00, 1.34)	0.05

[a]Model adjusted for age at randomization, randomization arm, center, sex, race, smoking status, ibuprofen use, and history of heart attack, stroke, hypertension, or diabetes.

Abbreviations: 95% CI, 95% confidence interval; BMI, body mass index; HR, hazard ratio

© 2023 Loomans-Kropp HA et al. *JAMA Network Open.*
