# Supplementary material for: Analysis of Body Mass Index in Early and Middle Adulthood and Estimated Risk of Gastrointestinal Cancer
Source: JAMA Netw Open. 2023 May 10;6(5):e2310002. doi: 10.1001/jamanetworkopen.2023.10002 (PMC10173015; doi:10.1001/jamanetworkopen.2023.10002)
Supplement: Supplement 2. — Data Sharing Statement [file jamanetwopen-e2310002-s002.pdf]

Data Sharing Statement

Loomans-Kropp. Analysis of Body Mass Index in Early and Middle Adulthood and Estimated Risk of Gastrointestinal Cancer. *JAMA Netw Open*. Published May 10, 2023. doi:10.1001/jamanetworkopen.2023.10002

Data
Data available: No

Additional Information
Explanation for why data not available: Data will be made available upon request to the corresponding author.
